# Oxidation of Cylindrospermopsin by Fenton Process: A Bench-Scale Study of the Effects of Dose and Ratio of H_2_O_2_ and Fe(II) and Kinetics

**DOI:** 10.3390/toxins13090604

**Published:** 2021-08-29

**Authors:** Matheus Almeida Ferreira, Cristina Celia Silveira Brandão, Yovanka Pérez Ginoris

**Affiliations:** Environmental Technology and Water Resources Postgraduation Program, Department of Civil and Environmental Engineering, University of Brasília, Brasilia 70910-900, Brazil; ferreira.m.a@outlook.com (M.A.F.); yovanka@unb.br (Y.P.G.)

**Keywords:** cylindrospermopsin removal, advanced oxidation processes, Fenton process

## Abstract

The cyanotoxin cylindrospermopsin (CYN) has become a significant environmental and human health concern due to its high toxicological potential and widespread distribution. High concentrations of cyanotoxins may be produced during cyanobacterial blooms. Special attention is required when these blooms occur in sources of water intended for human consumption since extracellular cyanotoxins are not effectively removed by conventional water treatments, leading to the need for advanced water treatment technologies such as the Fenton process to produce safe water. Thus, the present study aimed to investigate the application of the Fenton process for the degradation of CYN at bench-scale. The oxidation of CYN was evaluated by Fenton reaction at H_2_O_2_/Fe(II) molar ratio in a range of 0.4 to 4.0, with the highest degradation of about 81% at molar ratio of 0.4. Doubling the concentrations of reactants for the optimized H_2_O_2_/Fe(II) molar ratio, the CYN degradation efficiency reached 91%. Under the conditions studied, CYN degradation by the Fenton process followed a pseudo-first-order kinetic model with an apparent constant rate ranging from 0.813 × 10^−3^ to 1.879 × 10^−3^ s^−1^.

## 1. Introduction

Although natural aquatic ecosystems can be eutrophic as water bodies age and are filled in with sediments [1], human activities, such as agriculture, industry, and sewage disposal, can speed up the natural eutrophication of lentic systems by increasing the load of nutrients, resulting in anthropogenic eutrophication. The nutrients enrichment of an aquatic environment, mainly due to nitrogen and phosphorus, leads to a rapid growth of cyanobacteria, algae, and aquatic plants, which results in a shift in the biodiversity and ecosystem balance, compromising the water quality and various uses of water [2,3,4,5,6,7].

The fast growth of cyanobacteria in eutrophic waters, most frequently referred to as cyanobacterial bloom, can be harmful when the toxins produced by these organisms (cyanotoxins) reach dangerous concentrations to humans and animals. Dozens of genera and species of cyanobacteria are capable of producing toxins [8,9,10], which are classified as secondary metabolites.

The cylindrospermopsins (CYNs) are among the cyanotoxins of most significant health concern. These cyanotoxins are produced by species of various genera, including *Raphidiopsis* (previously *Cylindrospermopsis*), *Aphanizomenon*, *Dolichospermum* (previously *Anabaena*), *Lyngbya*, and *Umezakia*. Several CYN producing species are found in lakes, rivers, and drinking water reservoirs all over the globe [11], and it is known that CYN is harmful to both animal and human health, with hepatotoxic, nephrotoxic, immunotoxic, cytotoxic, and genotoxic effects [12,13,14,15].

Most cyanobacterial toxins such as microcystins (MCs), nodularins, and saxitoxins are primarily intracellular, and the dissolved fraction (extracellular toxin) is detected in the water body when cell lysis occurs. However, CYNs can also be released from viable cells into the aquatic environment during its life cycle [10]. While extracellular MCs represent less than 30% of total MCs (intracellular + extracellular toxin) [16], extracellular CYN is reported ranging from 50 to 90% of the total CYN [17,18,19]. In addition, extracellular CYN tends to be reasonably stable in surface water under sunlight irradiation, with a half-life of 11–15 days, although the presence of cell pigments can speed up the CYN oxidation [20].

As cyanotoxins are a threat to human health and their dissolved fractions are not effectively removed by conventional water treatment processes, special attention is necessary when cyanobacterial blooms occur in drinking water reservoirs [14,21,22,23,24].

In 1996 in Caruaru, Pernambuco state, Brazil, following the use of inadequately treated water from local eutrophic reservoirs, dozens of patients died after intravenous exposure to MCs during renal dialysis treatment [25,26]. After this tragic event, in 2000, the Brazilian Ministry of Health established the guideline values of 1 µg L^−1^ for MCs (mandatory) and 15 µg L^−1^ for CYN (recommended) [27]. Based on the studies by Humpage and Falconer [28], the CYN recommended guideline value was reduced to 1 µg L^−1^ in 2011 in Brazil [29], becoming mandatory only in 2021 [30] due to the increasing number of CYN occurrence reports.

Conventional drinking water treatments, in general, focus on cyanobacteria removal without compromising cell integrity to remove intracellular toxins, preventing lysis of cyanobacterial cells, thereby releasing toxins into the water. However, as high concentrations of extracellular CYN (usually ranging from <1 to 10 µg L^−1^ but occasionally up to 800 µg L^−1^ [31]) can be found in water bodies throughout the bloom development, even without cell lysis, the use of advanced water treatment process is a requirement to face the challenge of removing CYN, or any other extracellular cyanotoxin, in drinking water treatment.

In this context, advanced oxidative processes (AOPs) emerged as an important alternative to the effective removal and degradation of cyanotoxins in water treatment. These AOPS are based on the in situ generation of powerful and non-selective chemical oxidants such as hydroxyl and sulfate radicals, capable of reacting with a wide range of organic and inorganic pollutants.

Various AOPs can be applied to cyanotoxins removal from water, including O_3_, O_3_/UV, H_2_O_2_/UV, Fenton, photo-Fenton, electro-Fenton, and Fenton-like processes. Among them, the Fenton process is one of the most cost-effective [32,33,34,35] and has been gaining some attention due to its high performance, simplicity, short reaction times, and the lack of toxicity of the easy-to-handle reagents H_2_O_2_ and Fe(II) [36,37,38,39]. The advantages of the Fenton process compared to other AOPs can make this process more suitable for scale-up, especially in developing countries such as Brazil. In pre-existing water treatment plants, the Fenton process and iron-based coagulation can be easily combined in a single rapid-mix unit by adding H_2_O_2_.

In the Fenton process, the formation of hydroxyl radical involves several parallel and series reactions (Equations (1) to (7)) [40,41] that can be represented by a global reaction (Equation (8)) [42]. The kinetic rate constants for Equations (1) to (7) were reported elsewhere [40,41,43,44,45,46].

(1) $$\mathrm{Fe}\left(\mathrm{II}\right)+H_{2}O_{2}\to \mathrm{Fe}\left(\mathrm{III}\right)+{}^{•}\mathrm{OH}+{\mathrm{OH}}^{-}\;k\approx 70\;M^{-1}s^{-1}$$

(2) $$\mathrm{Fe}\left(\mathrm{III}\right)+H_{2}O_{2}\to \mathrm{Fe}\left(\mathrm{II}\right)+{\mathrm{HO}}_{2}^{•}+H^{+}\;k=0.001–0.01\;M^{-1}s^{-1}$$

(3) $${}^{•}\mathrm{OH}+H_{2}O_{2}\to {\mathrm{HO}}_{2}^{•}+H_{2}O\;k=3.3\times 10^{7}\;M^{-1}s^{-1}$$

(4) $${}^{•}\mathrm{OH}+\mathrm{Fe}\left(\mathrm{II}\right)\to \mathrm{Fe}\left(\mathrm{III}\right)+{\mathrm{OH}}^{-}\;k=3.2\times 10^{8}\;M^{-1}s^{-1}$$

(5) $$\mathrm{Fe}\left(\mathrm{III}\right)+{\mathrm{HO}}_{2}^{•}\to \mathrm{Fe}\left(\mathrm{II}\right)+O_{2}H^{+}\;k\leq 2\times 10^{3}\;M^{-1}s^{-1}$$

(6) $$\mathrm{Fe}\left(\mathrm{II}\right)+{\mathrm{HO}}_{2}^{•}+H^{+}\to \mathrm{Fe}\left(\mathrm{III}\right)+H_{2}O_{2}\;k=1.2\times 10^{6}\;M^{-1}s^{-1}$$

(7) $$2{\mathrm{HO}}_{2}^{•}\to H_{2}O_{2}+O_{2}\;k=8.3\times 10^{5}\;M^{-1}s^{-1}$$

(8) $$2\mathrm{Fe}\left(\mathrm{II}\right)+H_{2}O_{2}+2H^{+}\to 2\mathrm{Fe}\left(\mathrm{III}\right)+2H_{2}O$$

The degradation efficiency of the Fenton process depends on several parameters: pH, reaction time, temperature, initial concentration of pollutant, as well as reagents dosage and H_2_O_2_/Fe(II) molar ratio [47,48] since H_2_O_2_ and Fe(II) can also scavenge hydroxyl radicals (Equations (3) and (4)) if their concentrations are not optimized. The reagents dosage also reflects on the cost of the process and on the solids concentration, which can increase the iron sludge production and impair further treatment steps or discharge when high concentrations of Fe(II) are used [49].

Due to the potential to completely mineralize a variety of organic compounds, the Fenton process has been extensively studied over the past few decades. Numerous studies have been carried out applying the Fenton process for the removal of a diversity of pollutants, including phenol [50,51,52], bisphenol A [53,54], persistent organic pollutants [55], landfill leachate [56,57,58]. Concerning the removal of cyanotoxins, the studies have focused on MCs achieving degradation efficiency ranging from 18 to 100% [36,59,60,61,62,63].

To the best of our knowledge, no previously published study focused on CYN degradation by the traditional Fenton process. Only recently, however, few studies concerning the CYN oxidation by Fenton-like processes are available and reported a CYN degradation efficiency of around 90% [64,65]. The high CYN degradation efficiency obtained by using Fenton-like processes (Equation (2)), which are generally slower than Fenton process (Equation (1)), shows the promising potential for the CYN degradation by Fenton process. Additionally, the uracil ring, which is critical to CYN toxicity [66], is very susceptible to oxidation by hydroxyl radical [67,68]. Thus, the present study aimed to evaluate the oxidation (degradation) of CYN using Fenton process, at bench-scale, with emphasis on the effects of H_2_O_2_/Fe(II) molar ratio and the initial concentrations of H_2_O_2_ and Fe (II) on this cyanotoxin oxidation efficiency as well as the oxidation kinetics.

## 2. Results and Discussion

### 2.1. The Effect of H_2_O_2_/Fe(II) Molar Ration on CYN Degradation

**Figure 1:**
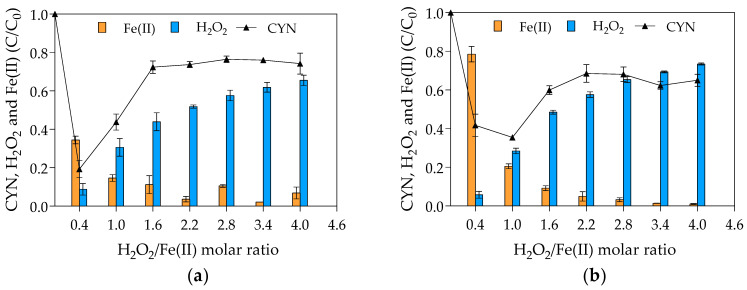
Relative residual concentration (C/C_0_) of CYN, H_2_O_2_ and Fe(II) for various H_2_O_2_/Fe(II) molar ratios. (**a**) First set of experiments conducted with initial H_2_O_2_ concentration of 25 μM and initial Fe(II) concentrations from 6.25 to 62.5 μM. (**b**) Second set of experiments conducted with initial Fe(II) concentration of 25 μM and initial H_2_O_2_ concentrations from 10 to 100 μM. (Initial CYN concentration ≈ 0.05 μM; initial pH = 5.0 ± 0.1; reaction time = 30 min. The values are averages of three replicates, and error bars indicate the standard deviation).

As pointed out in the Materials and Methods (Section 4.1), two sets of experiments were carried out to evaluate the effect of H_2_O_2_/Fe(II) molar ratio on the CYN degradation. In the first set, the initial H_2_O_2_ concentration was kept constant at 25 µM; and, in the second one the initial Fe(II) concentration was kept constant at 25 µM. Figure 1 presents the average results of the two sets.

The highest CYN degradation efficiency of 81% was achieved with an H_2_O_2_/Fe(II) molar ratio of 0.4 in the first set of experiments (Figure 1a) and 65% with H_2_O_2_/Fe(II) molar ratio of 1.0 in the second set of experiments. The effect of H_2_O_2_/Fe(II) molar ratio on the CYN oxidation presents a similar behavior in both sets of experiments, although the difference in the optimum H_2_O_2_/Fe(II) molar ratio. Regarding the blank synthetic water used to evaluate the degradation of CYN over the reaction time in the absence of Fenton reagents, less than 5% of CYN degradation was observed as expected because CYN standard is relatively stable in ultrapure water [20].

Comparing the CYN degradation efficiency at H_2_O_2_/Fe(II) molar ratio of 0.4 in the two sets of experiments, the lower degradation of 58% observed in the second set (Figure 1b) can be explained by the initial H_2_O_2_ and Fe(II) concentrations, which were 2.5 times lower in the second set than in the first set of experiments (see Figure 5). Additionally, comparing the relative residual Fenton reagents, while the relative residual H_2_O_2_ was approximately similar in both sets of experiments, the relative residual Fe(II) was at least two times lower in the first set (Figure 1b) than in the second set (Figure 1a), indicating that the increase in the Fenton reagents, especially Fe(II), increased the hydroxyl radical scavenging activity (see Equation (4)) and consequently the Fe(II) consumption.

**Figure 2:**
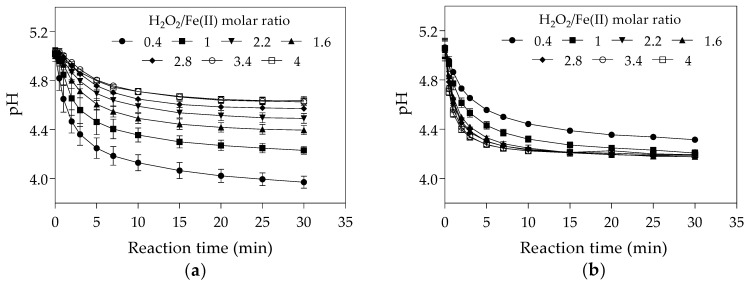
pH-time profile during Fenton oxidation under different H_2_O_2_/Fe(II) molar ratios. (**a**) The first set of experiments was conducted with initial H_2_O_2_ concentration of 25 μM and initial Fe(II) concentrations from 6.25 to 62.5 μM. (**b**) The second set of experiments was conducted with initial Fe(II) concentration of 25 μM and initial H_2_O_2_ concentrations from 10 to 100 μM. (The values presented are averages of three replicates, and error bars indicate the standard deviation).

The increase of the initial Fe(II) concentration may have led to a reduction in final pH as the hydrolysis of Fe(III) yielded in the Fenton reaction (Equation (1)) contributes to water acidification [69] (Figure 2a). The final pH at H_2_O_2_/Fe(II) molar ratio of 0.4 was lower in the first set of experiments (Figure 2a) than in the second set (Figure 2b). As higher degradation efficiencies of the Fenton process were reported at acidic conditions (pH values between 3 and 4) [61,70,71], the lowest final pH observed in the first set of experiments might have increased hydroxyl radical generation and consequently the CYN oxidation.

As can be seen in Figure 1, the degradation efficiency of CYN decreased when H_2_O_2_/Fe(II) molar ratio increased from 0.4 to 1.6 and remained constant at higher H_2_O_2_/Fe(II) ratios. The observed reduction in the degradation efficiency of CYN may be explained by the scavenging of hydroxyl radicals by the excess H_2_O_2_ (see Equation (3)) since the residual CYN concentrations showed a similar trend as the residual H_2_O_2_. Furthermore, the scavenging of hydroxyl radicals by H_2_O_2_ leads to the generation of hydroperoxyl radical that has a lower oxidation potential, 1.7 V (SHE), than the hydroxyl radical, 2.8 V (SHE) [72].

Concerning the high percentage of residual H_2_O_2_ (from 50 to 69%) and the low percentage of residual Fe(II) (from 1 to 9%) at H_2_O_2_/Fe(II) molar ratios above 1.0, higher degradation efficiency could be achieved over a long time since the regeneration of Fe(II) can be accomplished by residual H_2_O_2_ by the Fenton-like reaction (Equation (2)). However, the Fenton-like reaction is very slow (second-order rate constant of 0.001 to 0.01 M^−1^ s^−1^ [44]) in comparison with the Fenton main reaction (second-order rate constant of 70 M^−1^ s^−1^ [43]), Equation (1).

Despite the high reactivity of hydroxyl radical with Fe(II) in comparison with H_2_O_2_, with second-order rate constants respectively of 3.2 × 10^8^ and 3.3 × 10^7^ M^−1^ s^−1^ [45], low H_2_O_2_/Fe(II) molar ratios resulted in higher CYN degradation. However, the optimal H_2_O_2_/Fe(II) molar ratios observed in this study are in accordance with the molar ratios of the Fenton main (Equation (1)) and global (Equation (8)) reactions, which suggests that H_2_O_2_/Fe(II) molar ratios less than or equal to 1 might favor the hydroxyl radical generation. It must be emphasized that there is no consensus regarding the optimum H_2_O_2_/Fe(II) molar ratio since it also depends on specific conditions such as pH and type and concentration of pollutant. For MCs degradation by the Fenton process, the optimum H_2_O_2_/Fe(II) molar ratio reported is in a range of 0.7 to 15 [36,59,60,61].

Based on the CYN oxidation efficiencies, the value of 0.4 obtained from the first set of experiments was adopted as the optimum H_2_O_2_/Fe(II) molar ratio (25 µM H_2_O_2_ and 62.5 µM Fe(II)) for CYN degradation by the Fenton process for the conditions evaluated in this study.

### 2.2. The Effect of Initial H_2_O_2_ and Fe(II) Concentration on CYN Degradation

**Figure 3:**
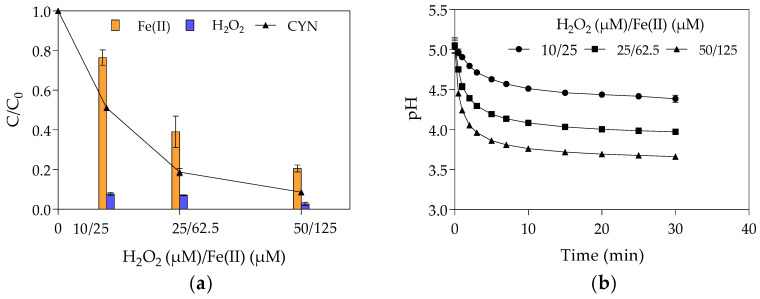
The effect of the Fenton reagent on CYN degradation by Fenton process at H_2_O_2_/Fe(II) molar ratio of 0.4. (**a**) relative residual concentration (C/C_0_) of CYN, H_2_O_2_, and Fe(II) and (**b**) pH-time profile. (Initial CYN concentration ≈ 0.05 μM. The values are averages of three replicates, and error bars indicate the standard deviation).

The effect of initial Fenton reagents concentrations on CYN degradation was evaluated at the optimal H_2_O_2_/Fe(II) molar ratio of 0.4 obtained from the first set of experiments in Phase 1 with initial H_2_O_2_ and Fe(II) concentrations of 0.4, 1.0, and 2.0 times their optimal concentrations (25 µM H_2_O_2_ and 62.5 µM Fe(II)). Figure 3 shows the relative residual concentration (C/C_0_) of CYN, H_2_O_2_, and Fe(II) and the pH-time profile for various initial concentrations of H_2_O_2_ and Fe(II).

The appropriate concentrations of H_2_O_2_ and Fe(II) are a key factor in enhancing the efficiency of the Fenton process. The analysis of reagent blank synthetic water showed that no significant CYN degradation (less than 10%) was observed at the highest concentrations of Fenton reagents when tested alone, that is, 100 µM H_2_O_2_ alone and 125 µM Fe(II) alone. Munoz and co-workers [64] also found no significant CYN degradation by heterogeneous Fenton-like reagents (H_2_O_2_ and Fe_3_O_4_-R400) when tested independently.

On the other hand, the Fe(II) and H_2_O_2_ interaction can lead to higher CYN degradation, even at low initial concentrations. As can be seen in Figure 3a, the CYN degradation was 49% with 10 µM H_2_O_2_ and 25 µM Fe(II), 81% with 25 µM H_2_O_2_ and 62.5 µM Fe(II), and 91% with 50 µM H_2_O_2_ and 125 µM Fe(II).

In addition, the relative residual H_2_O_2_ has remained almost constant, around 6%, while the relative residual Fe(II) decreased from 76% to 21% when the initial Fe(II) concentration increased from 25 to 125 µM (Figure 3a). As previously mentioned, this reduction in the relative residual Fe(II) was probably caused by the oxidation of Fe(II) to Fe(III) by hydroxyl radicals (Equation (4)).

Such behaviour suggests that, even at a fixed H_2_O_2_/Fe(II) molar ratio, the increase in the Fenton reagents led to an increase in the hydroxyl radical scavenging activity that can slow the rise in CYN degradation, as observed in Figure 3a. This explains why the increase from 25 µM H_2_O_2_ and 62.5 µM Fe(II) to 50 µM H_2_O_2_ and 125 µM Fe(II) only resulted in a rise of 10% in the CYN degradation, suggesting an asymptotic trend. This trend indicates that the CYN degradation by the Fenton process follows a reaction with an order greater than zero as the different initial concentrations of Fenton reagents resulted in different relative residual CYN after 30 min reaction (Figure 3).

Similarly, Park and co-workers [59], concerning the degradation of MC-LR by the Fenton process, also observed that increasing the H_2_O_2_ and Fe(II) concentration at a fixed H_2_O_2_/Fe(II) ratio increased the degradation efficiency until they reach a certain concentration above which all degradation efficiency increases appear to be insignificant.

The increase of the initial Fe(II) concentration diminished the final pH of the solution (Figure 3b), as also observed in the first set of experiments in Phase 1 (Figure 2a). As previously mentioned, acidic conditions may favour hydroxyl radical generation and consequently CYN oxidation. Although the positive effect of the high initial Fe(II) concentration on decreasing pH, the negative effect on hydroxyl radicals scavenging (Equation (4)) seems more significant.

### 2.3. Kinetic Assessment

During the Fenton process, H_2_O_2_ reacts with Fe(II) to produce mainly hydroxyl radical, hydroperoxyl radical and high-valent iron complexes, which can oxidize organic and inorganic compounds [48,73].

**Figure 4:**
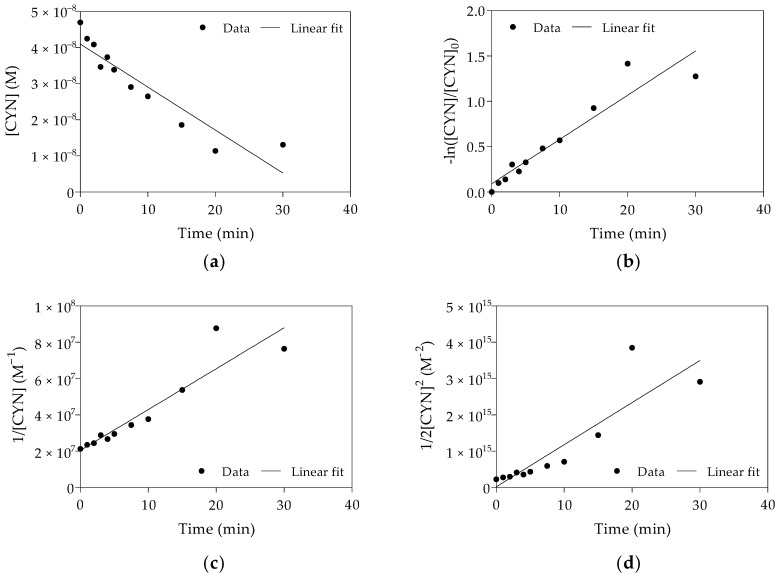
Linear plots and fitting of pseudo-zero-order (**a**), pseudo-first-order (**b**), pseudo-second-order (**c**) and pseudo-third-order (**d**) kinetic models to the experimental data obtained from Replicate 2. (Initial CYN concentration ≈ 0.05 µM; H_2_O_2_ = 25 µM; Fe(II) = 62.5 µM; initial pH 5.0 ± 0.1; 30 min reaction).

In this study, the kinetic experiments were performed with excess Fenton reagents. The H_2_O_2_ and Fe(II) concentrations at the optimal H_2_O_2_/Fe(II) molar ratio obtained from Phase 1 were respectively 500 and 1250 times greater than the initial CYN concentration. Due to the excess Fenton reagents, reaction order and rate constant were estimated from a pseudo reaction. The fitting of the kinetic models to the experimental data of one replicate is shown in Figure 4.

The kinetics parameters of the oxidation of CYN for each replicate are shown in Table 1. As shown in Figure 4 and based on R^2^ (Table 1), CYN oxidation by the Fenton process was best described by the pseudo-first-order kinetic model. The results obtained from all three replicates showed a similar trend concerning fitting this kinetic model to the experimental data.

As previously mentioned, to the best of our knowledge, no studies evaluating the CYN oxidation by Fenton process were published until the present. However, the oxidation of CYN and also MC-RR, MC-LR, anatoxin-a, and saxitoxin by heterogeneous Fenton-like process (H_2_O_2_/Fe_3_O_4_-R400) was reported by Munoz and co-workers [64]. The authors studied the oxidation of 1.2 µM of CYN, and 0.5 µM of MC-RR diluted in deionized water at pH 5 and with excess Fenton reagents (58.8 µM of H_2_O_2_ for CYN, 75.0 µM of H_2_O_2_ for MC-RR and fixed Fe_3_O_4_-R400 concentration of 863.8 µM). Under these conditions, the apparent pseudo-first-order rate constants of 7.4167 s^−1^ for CYN and 10.0167 s^−1^ for MC-RR were obtained.

The rate constant for CYN obtained by Munoz and co-workers [64] is significantly higher than that obtained herein (1.233 × 10^−3^ s^−1^, Table 1). Likewise, the rate constant for MC-RR found by the same authors [64] is also considerably greater than that reported by Zhong and co-workers (2.165 × 10^−3^ s^−1^) [61], who evaluated the degradation of 0.7 µM of MC-RR diluted in deionized-distilled water at pH 3 and using an excess of Fenton reagents (1500 µM of H_2_O_2_ and 100 µM of Fe(II)).

The results obtained by Munoz and co-workers [64] may be attributed to the nanocatalyst itself, which was specially designed and boosted for the heterogeneous Fenton-like oxidation [74]. As nanocatalysts have high surface areas and low diffusional resistance, they are more efficient than conventional heterogeneous catalysts [75].

The observed differences in the apparent pseudo-first-order rate constants reflect the different radical generations in each process. It should be pointed out that higher apparent rate constants may indicate higher hydroxyl radical concentration since the hydroxyl radical concentration is incorporated in the apparent rate constant and/or higher susceptibility to hydroxyl radical oxidation.

Despite these differences, the apparent rate constant for CYN degradation found in the present study is in the same order of magnitude as that (4 × 10^−3^ s^−1^) reported by Chen and co-workers [67] obtained by using UV-TiO_2_ photocatalysis under the following conditions: 2.4 µM of initial CYN, 313 µM of TiO_2_, O_2_ saturation, and 350 nm irradiation with an intensity of about 1.12 × 10^16^ photons s^−1^ cm^−3^.

## 3. Summary and Conclusions

The degradation of CYN standard in ultrapure water was investigated by means of the Fenton process. The results showed that the CYN removal increased as the H_2_O_2_/Fe(II) molar ratio decreased. Within the range of H_2_O_2_/Fe(II) molar ratio tested (0.4 to 4.0), the highest CYN degradation of 81% was obtained when H_2_O_2_/Fe(II) molar ratio was 0.4 (25 µM H_2_O_2_ and 62.5 µM Fe(II)).

The increase of the dosage of Fenton reagents (50 µM H_2_O_2_ and 125 µM Fe(II)) at the optimal H_2_O_2_/Fe(II) molar ratio of 0.4 resulted in an increase of the CYN oxidation efficiency to 91%. The CYN oxidation by the Fenton process followed a pseudo-first-order kinetic model with an apparent rate constant of 1.233 × 10^−3^ s^−1^.

Based on the results herein obtained, the Fenton process was effective for the removal of CYN from ultrapure water. Thus, the Fenton process seems to be a promising alternative for the CYN removal in drinking water treatment that could be easily implemented in full-scale worldwide since Fenton process is quite simple and highly cost-effective. However, further studies are necessary to evaluate matrix effects and analyze the feasibility and applicability of the Fenton process to treat natural water with high concentrations of CYN and much higher concentrations of hydroxyl radical scavenging compounds such as natural organic matter.

## 4. Materials and Methods

### 4.1. Chemicals

Cylindrospermopsin standard (purity > 95%) was purchased from Eurofins/Abraxis (Eurofins/Abraxis, Warminster, PA, USA). Methanol (99.9% HPLC grade), Ferrozine (97%), and peroxidase from horseradish (type II) were obtained from Sigma-Aldrich (Sigma-Aldrich, São Paulo, SP, Brazil). Acetic acid glacial (99.7% HPLC grade) was purchased from J.T Baker (J. T. Baker, Brazil). Hydroxylamine hydrochloride (96%) and ammonium hydroxide (27% *v*/*v*) were purchased from Synth (Synth, Diadema, SP, Brazil). Ammonium acetate (97%), N,N-Diethyl-p-phenylenediamine sulfate salt (98%), sodium phosphate dibasic (98%), sodium phosphate monobasic (98%), and hydrogen peroxide (35% *v*/*v*) were obtained from Neon (Neon, Suzano, SP, Brazil). Sodium sulfite (98%), sulfuric acid (98% *v*/*v*), hydrochloric acid (36.5% *v*/*v*), iron (II) sulfate heptahydrate (99%) and iron (III) chloride hexahydrate (97%) were purchased from Dinâmica (Dinâmica, Indaiatuba, SP, Brazil).

All solutions used in the experiments were prepared using ultrapure water (Milli-Q Reference water purification system, C79625, Merck Millipore, Darmstadt, Hesse, Germany).

### 4.2. Experimental Setup

Fenton experiments were performed in 250 or 500 mL borosilicate glass beaker batch reactors at room temperature (23 to 25 °C). The oxidation experiments were carried out with a CYN solution with initial concentration (C_0_) of 0.05 µM prepared by diluting a CYN stock solution (0.12 µM) with ultrapure water. This solution will be referred to as “synthetic water”. Oxidation reactions were started by adding to the synthetic water predetermined amounts of Fe(II), immediately followed by the addition of H_2_O_2_, under vigorous magnetic stirring. After the desired reaction time, a sodium sulfite solution (2 times stoichiometric excess of Na_2_SO_3_ to H_2_O_2_ [76]) was added to quench the residual H_2_O_2_, stopping the generation of hydroxyl radicals. The Fe(II), H_2_O_2_, and sodium sulfite stock solutions were always prepared fresh.

All of the experiments were carried out in triplicates. The synthetic water initial pH was adjusted to 5.0 ± 0.1 with 50 mM H_2_SO_4_ and measured with a pH probe (Scientific Orion 3 Star portable pH meter, Thermo Fisher Scientific, Waltham, MA, USA).

**Figure 5:**
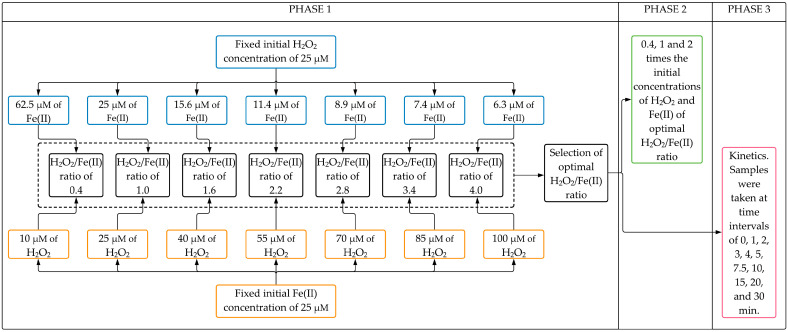
Flowchart of the experimental setup.

The experimental setup can be divided into three phases (Figure 5).

Phase 1 aimed to evaluate the effect of H_2_O_2_/Fe(II) molar ratio on the CYN degradation after 30 min Fenton reaction. Seven H_2_O_2_/Fe(II) molar ratios (0.4, 1.0, 1.6, 2.2, 2.8, 3.4, and 4.0) were evaluated in two sets of experiments. In the first set, experiments were conducted with a fixed initial H_2_O_2_ concentration of 25 µM with initial Fe(II) concentrations ranging from 6.25 to 62.5 µM (see Phase 1 blue boxes in Figure 5). After that, similar experiments were performed in the second set, keeping fixed the initial Fe(II) concentration at 25 µM with initial H_2_O_2_ concentrations ranging from 10 to 100 µM (see Phase 1 orange boxes in Figure 5). The optimal H_2_O_2_/Fe(II) molar ratio was chosen based on the highest CYN degradation obtained from both sets of experiments. Additionally, In Phase 1, blank synthetic water was used to evaluate the degradation of CYN over the reaction time in the absence of Fenton reagents.

In Phase 2, experiments were conducted to evaluate the effect of the initial concentrations of H_2_O_2_ and Fe(II) on CYN degradation after a 30 min reaction time. These oxidation experiments were performed at initial concentrations of H_2_O_2_ and Fe(II) 0.4, 1, and 2 times the initial concentrations of the optimal H_2_O_2_/Fe(II) molar ratio obtained from Phase 1. Additionally, in Phase 2, the degradation of CYN over the reaction time was also evaluated using Fenton reagents (H_2_O_2_ and Fe(II)) separately at the highest concentrations tested in this study.

In Phase 3, oxidation kinetics of CYN was investigated in Fenton experiments conducted at the optimal H_2_O_2_/Fe(II) molar ratio as determined in Phase 1. Samples were taken at time intervals of 0, 1, 2, 3, 4, 5, 7.5, 10, 15, 20, and 30 min.

### 4.3. Detection and Quantification of H_2_O_2_

The H_2_O_2_ concentration was measured using the horseradish peroxidase (POD)- N,N-Diethyl-p-phenylenediamine (DPD) photometric method as described by Hahn and co-workers [77]. Hydrogen peroxide was measured immediately after the reaction time, withdrawing a sample from the reactor before adding the sodium sulfite solution.

In the analytical routine, a 13.5 mL aliquot of the oxidized synthetic water was transferred to a 50 mL beaker and, under magnetic stirring, 1.5 mL of a solution of 0.5 M Na_2_HPO_4_ plus 0.5 M NaH_2_PO_4_ was added. Right after that, 25 µL of 38.12 mM DPD solution (prepared in 50 mM H_2_SO_4_) and 25 µL of 100 units mL^−1^ POD were added. The mixture was allowed to react for 40 s and then was transferred to a 5 cm path length quartz cuvette cell. The absorbance was measured at a wavelength of 551 nm using a UV-Vis spectrophotometer (DR 5000, Hach, Loveland, CO, USA).

For H_2_O_2_ quantification, a 6-point calibration curve encompassing H_2_O_2_ concentrations over the range of 0 to 10.29 µM was used. Samples were diluted using ultrapure water when their concentration was higher than the range of the calibration curve. The limit of detection (LoD) of 0.06 µM was determined according to Eurachem guidelines [78].

### 4.4. Detection and Quantification of Fe(II) and Total Iron

The concentrations of Fe(II) and total iron were measured by the ferrozine photometric method [79]. Similar to the hydrogen peroxide, residual Fe(II) and total iron were measured immediately after the reaction time.

Initially, to quantify residual Fe(II), a 0.3 mL of a 10 mM ferrozine solution (prepared in 0.1 M ammonium acetate) was added to a 2.7 mL sample of the oxidized synthetic water to form the Fe(II)-ferrozine complex whose absorbance can be measured at a wavelength of 562 nm using a UV-Vis spectrophotometer (DR 5000, Hach) employing 1 cm path length quartz cuvette cell.

Following the analytical routine, to quantify the total iron present, 0.45 mL of 1.4 M hydroxylamine hydrochloride (prepared in 2 M HCl) was added to a 2.4 mL aliquot of Fe(II)-ferrozine complex solution in order to reduce the Fe(III) to Fe(II) species. This mixture was allowed to react for ten minutes, and then 0.15 mL of 10 M ammonium acetate was added, and the absorbance of the resultant solution was also measured at a wavelength of 562 nm. The Fe(III) concentration was calculated as the difference between total iron and Fe(II) concentrations.

For Fe(II) and total iron quantifications, a 6-point calibration curve encompassing Fe(III) concentrations over the range of 0 to 6.72 µM was used. Samples were diluted using ultrapure water when their concentration was higher than the range of the calibration curve. The LoD of 0.61 µM for Fe(II) and 0.64 µM for total iron were determined according to Eurachem guidelines [78].

### 4.5. Detection and Quantification of CYN

Cylindrospermopsin was determined by high-performance liquid chromatography (Agilent 1200 Series, Agilent Technologies, Palo Alto, CA, USA) coupled to mass spectrometry (3200 QTRAP, Sciex, Toronto, ON, Canada) with an electrospray ion source operating in the positive mode, using N_2_ as curtain (20 psi) and source gas (40 psi) under a capillary spray voltage of 5 kV at 450 °C.

Separation from matrix interferents was performed using a Kromasil 100-5 C18 column (100 × 4.6 mm, 5 μm, Akzo Nobel, Bohus, Sweden), coupled to its corresponding guard column (3.0 × 4.6 mm, 5 μm) at room temperature (19 to 21 °C), using 0.15% (*v*/*v*) acetic acid solutions prepared in ultrapure water (A) and methanol (B) as mobile phase, at a flow rate of 0.55 mL min^−1^. Gradient elution was achieved by increasing B from 10% (initial condition) to 30% in 0.5 min, to 90% in 7.5 min, held B constant for 2 min, and returning to the initial condition in 2 min. Under these conditions, CYN eluted at approximately 4.6 min.

For MS acquisition, a declustering potential of 56 V was applied to the orifice to prevent the ions from clustering together. Multiple reaction monitoring (MRM) was used for CYN detection and quantification through the monitoring of three precursor-to-product ion transitions. The most intense one, at m/z 416.1 to 194.3 (43 eV CE), was used for quantification, while transitions at m/z 416.1 to 336.1 (29 eV CE) and 416.1 to 176.2 (45 eV CE) were used for confirmation purposes.

Quantification was performed by external calibration using a 6-point analytical curve encompassing CYN concentrations over the range of 2.41 nM to 0.12 µM. The LoD of 0.24 nM was determined according to Eurachem guidelines [78]. Since the LoD was quite low, sample extraction and extract concentration were not necessary for quantification of CYN.

## Figures and Tables

**Table 1:** Kinetics parameters of CYN oxidation by Fenton process. (Initial CYN concentration ≈ 0.05 µM; H_2_O_2_ = 25 µM; Fe(II) = 62.5 µM; initial pH 5.0 ± 0.1; 30 min reaction).

Kinetic Model	Replicate 1			Replicate 2 ^b^			Replicate 3			Average		
	k ^a^	T_1/2_ (min)	R^2^	k ^a^	T_1/2_ (min)	R^2^	k ^a^	T_1/2_ (min)	R^2^	k ^a^	T_1/2_ (min)	R^2^
Zero	1.925 × 10^−11^	19.1	0.76	1.987 × 10^−11^	19.7	0.87	3.799 × 10^−11^	11.9	0.90	2.570 × 10^−11^	16.9	0.84
First	1.007 × 10^−3^	11.5	0.80	0.813 × 10^−3^	14.2	0.90	1.879 × 10^−3^	6.1	0.98	1.233 × 10^−3^	10.6	0.89
Second	6.435 × 10^4^	5.9	0.73	3.748 × 10^4^	9.5	0.87	13.390 × 10^4^	2.3	0.87	7.858 × 10^4^	5.9	0.82
Third	4.978 × 10^12^	2.6	0.60	1.934 × 10^12^	5.9	0.79	13.460 × 10^12^	0.6	0.72	9.219 × 10^12^	3.0	0.70

^a^ k is the apparent pseudo-zero-order rate constant (M s^−1^), pseudo-first-order rate constant (s^−1^), pseudo-second-order rate constant (M^−1^ s^−1^), pseudo-third-order rate constant (M^−2^ s^−1^); ^b^ data showed in Figure 4.
